# Does plaque morphology truly not matter? Reply

**DOI:** 10.1093/eurheartj/ehac765

**Published:** 2022-12-30

**Authors:** Lauri Holmström, Samuli Juntunen, Juhani Junttila

**Affiliations:** Research Unit of Internal Medicine, Medical Research Center Oulu, University of Oulu and Oulu University Hospital, PO Box 5000, 90014 Oulu, Finland; Research Unit of Internal Medicine, Medical Research Center Oulu, University of Oulu and Oulu University Hospital, PO Box 5000, 90014 Oulu, Finland; Research Unit of Internal Medicine, Medical Research Center Oulu, University of Oulu and Oulu University Hospital, PO Box 5000, 90014 Oulu, Finland

**This commentary refers to ‘Plaque histology and myocardial disease in sudden coronary death: the Fingesture study’, by L. Holmström *et al*., https://doi.org/10.1093/eurheartj/ehac533 and the discussion piece ‘Does plaque morphology truly not matter?’, by W. Wu and D.-M. Zhang, https://doi.org/10.1093/eurheartj/ehac756.**

We thank Wu and Zhang for their interest in our work and thoughtful discussion. They raised interesting aspects related to our recent work on coronary artery plaque morphology and myocardial disease in sudden cardiac death (SCD) due to coronary artery disease (CAD).^1^ While our study aimed to investigate the prevalence of acute plaque complications in SCD, Wu and Zhang discussed the potential significance of previous and healed plaque ruptures/erosions.

The authors noticed that a high proportion of SCD victims with stable plaques in our study had old infarct scars at autopsy (56.6%) and speculated that this may be related to previous coronary plaque complications and consequent silent myocardial infarctions (MIs). Silent MI is generally characterized as an MI in the absence of major or with unrecognized symptoms. Silent MIs are indeed common findings in SCD, and in our previous study, we found that 42% of SCD associated with undiagnosed CAD had evidence of MI at autopsy (i.e. silent MI).^2^ However, in the present study, we also included SCD victims with previously diagnosed CAD, and hence, we cannot precisely evaluate which proportion of old infarct scars at autopsy was due to silent MIs. Yet these infarct scars likely had a significant role in the incidence of SCD.

The authors also discussed the plaque healing process after acute complications, which leads to a layered plaque and a narrowing coronary lumen.^3^ They speculate that previously healed plaque complications have had a significant contribution to the development of severely obstructed (>75%) and stable coronary arteries in our SCD victims. They support this hypothesis by the finding that SCD victims with stable plaques had a higher prevalence of >75% coronary stenoses than SCD victims with acute plaque complications. We agree that previously healed plaque complications have probably contributed to the development of significant coronary stenoses, but comparing the prevalence of >75% stenosis is not reasonable given that we excluded the SCD victims with stable plaques and <75% coronary stenosis at autopsy. Furthermore, our study was focused on the immediate causes of SCD and the authors are discussing long-term changes in plaques. Therefore, the view of the issue at hand is different.

SCD is not a consequence of single coronary events, but rather a result of an interplay between an underlying substrate (myocardial disease), acute triggers (e.g. acute ischaemia), and maintainers. While the prevalence of acute plaque complications was less than half in our study, we agree that coronary plaque complications may have a role in the occurrence of SCD also beyond the acute phase, given that such complications are followed by arterial narrowing or myocardial scars, which create a substrate for future arrhythmias. The results of our study have significance in the evaluation of the mechanism and incidence of SCD during acute MI, but do not explain the mechanism of CAD which has been extensively studied in prior studies.

## Data Availability

The data underlying the original article cannot be shared publicly due to potentially identifiable nature.

## References

[1] Holmström L , Juntunen S, Vähätalo J, Pakanen L, Kaikkonen K, Haukilahti A, et al Plaque histology and myocardial disease in sudden coronary death: the Fingesture study. Eur Heart J 2022;43:4923–4930. 10.1093/eurheartj/ehac533 PMC9748531 36172703

[2] Vähätalo JH , Huikuri HV, Holmström LTA, Kenttä TV, Haukilahti MAE, Pakanen L, et al Association of silent myocardial infarction and sudden cardiac death. JAMA Cardiol 2019;4:796–802. 31290935 10.1001/jamacardio.2019.2210 PMC6624824

[3] Vergallo R , Crea F. Atherosclerotic plaque healing. N Engl J Med 2020;383:846–857. 32846063 10.1056/NEJMra2000317

